# Fracture resistance of molars with class II MOD cavities restored with bulk-fill, no-cap flowable bulk-fill, and conventional resin composite restorative systems after 6-months water storage

**DOI:** 10.1186/s12903-025-05951-1

**Published:** 2025-05-20

**Authors:** Ramy Ahmed Wafaie, Basma Ahmed, Salah Hasab Mahmoud

**Affiliations:** 1 Conservative Dentistry Department, Faculty of Oral and Dental Medicine, Delta University for Science and Technology, Gamasa, Egypt; 2 Conservative Dentistry Department, Faculty of Dentistry, Mansoura University, Mansoura, Egypt

**Keywords:** Fracture resistance, Class II cavities, Resin composite, Bulk-fill, Flowable bulk-fill

## Abstract

**Background:**

Bulk-fill resin composites are innovative materials, developed to simplify the placement of direct composite restorations especially in large cavities. Recently, a new class of flowable bulk-fill resin composite is claimed to be placed without final capping layer. Thus, this study aimed to assess and compare the fracture resistance of molars with Class II MOD cavities restored with different types of resin composite restorative systems after 6-month water storage.

**Methods:**

One-hundred sound mandibular molars were assigned randomly into 5 groups (*n* = 20). The teeth in the first group were left intact and tested as unprepared positive control (group I), while teeth in the remaining four groups received Class II MOD cavities. One of the prepared groups was left unrestored and tested as negative control (group II). The remaining three groups were restored as follows; group III: bulk-fill resin composite/Filtek One Bulk Fill (3 M Oral Care), group IV: no-cap flowable bulk-fill resin composite/G-aenial bulk injectable (GC), and conventional resin composite/Neo Spectra ST LV (Dentsply Sirona). Half of the specimens of each group (*n* = 10) was stored in distilled water at 37 °C for 24 h and then thermocycled 5000 times between 5 °C ± 2 °C and 55 °C ± 2 °C (immediate), while the other half was stored for 6 months before thermocycling (delayed). All specimens were loaded occlusally in a universal testing machine using a metal sphere that contacted the teeth at the cuspal inclines until fracture occurred. The results were analyzed by 2-way ANOVA and Tukey HSD post-hoc multiple comparison tests. The level of significance was set at *p* < 0.05.

**Results:**

Regardless of storage time, intact molar teeth showed significantly higher fracture resistance mean values than did the other tested groups (*p* < 0.05). The groups restored with bulk-fill and conventional resin composites showed higher mean values compared to the group restored with no-cap flowable bulk-fill resin composite (*p* < 0.05). The prepared unrestored group exhibited the lowest fracture resistance mean values (*p* < 0.05). Statistically significant differences were observed when comparing immediate and delayed restored groups (*p* < 0.05).

**Conclusions:**

The fracture resistance of Class II MOD cavities restored with bulk-fill or conventional resin composites was superior to those restored with no-cap flowable bulk-fill resin composite. Moreover, 6-month water storage had a deleterious effect on the fracture resistance of the restored molar teeth.

## Background

Over the last years, patients’ demands have changed from tooth restoration only to esthetics and maintenance of function [1] Resin composites have become the most preferred restorative materials by dental practitioners for the treatment of posterior teeth especially after the gradual phase-down of dental amalgam [2, 3]. These materials possess several advantages including satisfactory adhesion to enamel and dentin, conservation of the remaining tooth structure without the need for extensive means of retention, superior physical properties, availability of various shades and translucencies, and biocompatibility [4, 5]. It has been stated that posterior composite restorations showed excellent clinical performance with annual failure rates ranging from 1 to 3% [6]. Rodolpho et al. [7] also reported satisfactory success rates of Class I and II composite restorations after 33-year clinical evaluation.

Despite all these features and the developments experienced in recent years, the main challenges encountered when using direct resin composites are polymerization shrinkage and the associated stresses [8, 9]. These generated stresses can result in cuspal deflection, tooth cracking or marginal deficiencies which in turn may cause marginal staining, microleakage, gap formation, postoperative sensitivity, and secondary caries [10, 11]. Shrinkage stresses are influenced by several factors such as cavity configuration (C-factor), restorative material properties, and restoration application technique [12]. Previous systematic reviews [13, 14] reported that the most common reasons for the replacement of posterior composite restorations were fracture and secondary caries.

Conventional resin composites are usually applied in increments of up to 2 mm in thickness which are photo-cured separately. This incremental filling protocol is considered the standard technique to ensure high depth of cure and proper monomer to polymer conversion in addition to tackle polymerization shrinkage stresses [15, 16]. However, this technique has some drawbacks including the possibility of internal flexure on the preparation walls, intermediate layer contamination, and high risk of trapping voids between layers [17]. Hence, more attention has to be paid during placement of each layer to guarantee good adaptation and bonding between increments especially in restoring deep and wide cavities [18–20].

The goal of simplifying restorative procedures in a way that does not endanger the quality but rather enhances the results has paved the way for the development of bulk-fill resin composite materials presenting several clinical benefits [3, 11, 21, 22]. Manufacturers claim that these bulk-fill materials can be cured to a depth of 4–5 mm reducing the time required to place the restoration by up to 30% [23–25]. By shortening the treatment procedures, bulk-fill resin composites may aid in increasing patient and clinician comfort, ensuring higher clinical success as proven by Çağırır Dindaroğlu and Yılmaz [26]. The incorporation of stress-relaxant polymerization modulators, stress relievers, and high-molecular weight monomers in these materials minimizes stresses formation in the restoration to 1.13 MPa [9, 27]. Moreover, increasing the translucency of the material by using alternative photoinitiators, reducing filler load, and improving filler particle size enhance the polymerization depth by increasing light transmission to deeper areas. Their smooth consistency results in higher internal adaptation to the cavity walls and floor, thus better marginal integrity and low postoperative sensitivity [28–30]. Previous reports [16, 31] concluded that bulk-fill restorative technique showed acceptable clinical performance compared to the incremental filling technique after 3 and 5 years of clinical evaluations.

Like conventional composites, current commercial bulk-fill resin composites can be classified according to their viscosity into sculptable (high viscosity) and flowable (low viscosity) [32]. High viscosity bulk-fill composite has higher filler content than flowable type, and thus can replace dentin and enamel in a single increment without any veneering layer [22, 33, 34]. Several improvements have been done to enhance the performance of bulk-fill composites compared to their first predecessors. 3 M Oral Care released Filtek One Bulk Fill with modified chemical composition incorporating both patented addition fragmentation monomer (AFM) and aromatic urethane dimethacrylate (AUDMA) in order to reduce polymerization shrinkage stresses [35]. On the other hand, flowable type was only used in the proximal box or as a dentin replacement material that required capping layer of conventional composite due to its low surface hardness and modulus of elasticity [18, 36]. To overcome these limitations, a new flowable bulk-fill composite (G-aenial bulk injectable, GC) was introduced to the dental market with satisfying wear resistance and flexural strength in addition to lower polymerization shrinkage. This material can be placed in one layer up to 4 mm without final composite coverage [37].

Restorative procedures cause loss of enamel continuity and increase the susceptibility of teeth to fracture [38]. The main reasons for decreasing the fracture resistance of tooth/restoration complex are increasing the isthmus width and the gingival depth of preparation in addition to loss of marginal ridges [39]. These can result in microscopic fractures of the restoration margins, bulk fracture of the restoration or fracture of cusps especially in teeth with wide Class II cavities when subjected to repeated occlusal loading [20, 40, 41]. Therefore, the success of any restorative material is mainly attributed to its ability to strengthen the remaining tooth structure in order to withstand the oral masticatory forces. Several studies [20, 42–44] reported contradictory results when comparing the fracture resistance of MOD cavity preparations restored with conventional and bulk-fill resin composites. According to the authors’ knowledge, studies evaluating flowable bulk-fill composite without capping layer are lacking. Therefore, this study aimed to assess and compare the fracture resistance of molars with Class II MOD cavities restored with bulk-fill, no-cap flowable bulk-fill, and conventional resin composite restorative systems after 6-month water storage. The null hypothesis tested was that fracture resistance of molar teeth with Class II MOD cavities would not be influenced by the type of resin composite restorative system or the time of storage.

## Methods

### Restorative materials

Three commercially available resin composite restorative materials with their corresponding adhesive systems (Table 1) were employed in this study as follows; bulk-fill resin composite (Filtek One Bulk Fill/Single bond universal, 3 M Oral Care, St. Paul, MN, USA), no-cap flowable bulk-fill resin composite (G-aenial bulk injectable/ G-Premio Bond, GC, Tokyo, Japan), and conventional resin composite (Neo Spectra ST LV/Prime&Bond Universal, Dentsply Sirona GmbH, Konstanz, Germany). They were used in accordance with the manufacturers’ instructions. Light polymerization was performed using a light emitting diode (LED) curing unit (Elipar S10, 3 M Oral Care) with a wave length between 430 and 480 nm and a light intensity 1200 mW/cm2. The curing unit has a built-in radiometer in order to monitor the irradiance.

**Table 1:** Materials used in the study

Restorative system	Type	Manufacturer	Composition	Batch NO.
Filtek One Bulk-Fill	Nanofilled Bulk-fill resin composite	3 M Oral Care	Silane treated ceramic, silane treated silica and zirconia, diurethane dimethacrylate, UDMA, DDDMA, EDMAB, YbF3, water.	N604387
Single bond universal	Universal Adhesive		MDP phosphate monomer, dimethacrylate resin, HEMA, filler, ethanol, water, initiators, silane, vitrebond copolymer.	517571
G-aenial bulk injectable	Flowable Nanohybird Bulk-fill resin composite	GC	Bis-EMA, bismethacrylate, dimethacrylate, UV-light absorber, UDMA.	2009072
G-Premio Bond	Universal Adhesive		MDP, 4-MET, MDTP, BHT, dimethacrylate monomer, acetone, water, photoinitiator, silica fillers.	2310064
Neo Spectra ST LV	Nanoceramic resin composite	Dentsply Sirona	Spherical pre-polymerized SphereTEC fillers, non-agglomerated barium glass and ytterbium fluoride, highly dispersed methacrylic polysiloxane nano-particles.	2305000755
Prime & Bond Universal	Universal Adhesive		Bi- and multifunctional acrylate, phosphoric acid modified acrylate resin, initiator, stabilizer, isopropanol, water, PENTA, MDP.	2002000692

### Specimens and their preparation

A total of one-hundred sound freshly extracted human mandibular molars were collected from healthy individuals from the Department of Oral Surgery, Faculty of Oral and Dental Medicine, Delta University for Science and technology, Gamasa, Egypt. The Faculty Research Ethics Committee approved this research work under protocol number 0240121001. Any calculus, residual plaque, and soft tissue remnants were removed using a manual scaler (Zeffiro, Lascod, Florence, Italy). Teeth were stored in 0.5% chloramine-T as a disinfectant solution for 48 h and then cleaned using a rubber cup and fine pumice water slurry. All teeth were approximately similar in dimensions with a buccolingual width of 10.5 ± 0.5 mm as measured by digital caliber. A binocular stereomicroscope (SZ TP, Olympus, Tokyo, Japan) was used for ensuring that the selected teeth were free from caries and cracks. Finally, teeth were preserved in distilled water at 37 °C ± 1 °C using an incubator (BTC, BioTech Company, Cairo, Egypt) in order to avoid their dehydration during the test procedures [9, 38, 45].

For simulation of periodontal ligament, the roots of the teeth were covered with 0.2–0.3 mm layer of vinyl-polylsiloxane impression material (Kromopan, Lascod S.P.A., Florence, Italy). Then, the teeth were fixed in autopolymerizing acrylic resin blocks up to 2 mm below the cementoenamel junction (C.E.J) [33, 38, 44, 46]. Teeth were assigned randomly into five main groups (*n* = 20) as presented in Table 2.

**Table 2:** Study groups and their description

Group name	Group description	Filling procedure
Group 1	Intact teeth (positive control)	None
Group 2	Class II MOD cavities prepared but unrestored (negative control)	None
Group 3	Class II MOD cavities prepared and restored with bulk-fill resin composite (Filtek One Bulk Fill)	Bulk-fill
Group 4	Class II MOD cavities prepared and restored with no-cap flowable bulk-fill resin composite (G-aenial bulk injectable)	Bulk-fill
Group 5	Class II MOD cavities prepared and restored with conventional resin composite (Neo Spectra ST LV)	Incremental-fill

A single operator prepared standardized Class II MOD cavities using a flat-ended, straight fissure diamond instrument (836.HP.027; Komet, Brasseler, Lemgo, Germany) in a high-speed handpiece (Dentsply Sirona GmbH, Konstanz, Germany) under copious air-water cooling. The cutting instrument was replaced after every four preparations in order to maintain the cutting efficiency. To obtain standardized depth and width for all preparations, the handpiece was fixed in a specially designed device manufactured at Production Engineering and Mechanical Design Department, Faculty of Engineering, Mansoura University [45, 46]. The isthmus width of the preparation was 2/3 of the intercuspal distance. The width of the proximal boxes was also 2/3 of the total buccolingual distance. The depth of the pulpal floor was 4 mm from the central groove. The buccal and lingual walls were prepared parallel to each other with 90° cavosurface angle. Finishing of the prepared cavities was performed using extra-fine diamond instruments (835KREF.314.012, Komet) to obtain rounded line and point angles. A digital caliper was used for measuring and verifying cavity dimensions [43].

### Restorative procedures

In order to reestablish the proximal contours, a circumferential metal matrix/band (Omni-Matrix™, Ultradent Products, South Jordan, UT, USA) was adapted around each prepared tooth and supported externally by using low-fusing compound to ensure optimum adaptation to all cavity margins [33, 47].

Selective enamel etching technique was used as a standard protocol for all restored groups by applying 37% phosphoric acid gel (N-Etch, IvoclarVivadent AG, Schaan, Liechtenstein) on the enamel margins for 15–20 s. The preparation was then thoroughly rinsed by water for 10 s and gently dried with oil-free air. Each universal adhesive was applied to enamel and dentin using microbrush, scrubbed for 20 s, gently air-dried for 5 s to evaporate the solvent, and then light polymerized for 10 s following the manufacturers’ instructions. A titanium-coated hand instrument (curved paddle “LRT,” Nordent, Bonnie Lane, USA) was used for placing and sculpting of resin composite restorative materials. For groups III and IV, the prepared cavities were restored with Filtek One Bulk Fill and no-cap flowable bulk-fil resin composites in one bulk increment (4 mm) respectively and light-cured for 10 s. While for group V, conventional resin composite (Neo Spectra ST LV) was applied using incremental layering technique with 2 mm thickness for each increment. Each layer was light-cured separately for 10 s. Finally, the proximal surfaces of all restorations were additionally light-cured for 10 s from the buccal and lingual aspects after removing the matrix band in order to ensure adequate curing of the resin composite margins. Finishing and polishing procedures were accomplished using high-speed diamond finishing instruments (4092.314, Komet) under copious air-water cooling followed by flexible discs (Sof-Lex XT Pop On, 3 M Oral Care) and rubber polishing points (OneGloss, Shofu, Kyoto, Japan).

Half of the specimens of each group (*n* = 10) was stored in distilled water in an incubator at 37 °C for 24 h (immediate). Then, they were subjected to thermal cycling treatment that comprised 5000 cycles between 5 °C ± 2 °C and 55 °C ± 2 °C with dwell time of 30 s and transfer time of 2 s from one bath to the other [38]. The other half of the specimens was stored for 6 months before subjected to thermal cycling (delayed).

### Testing

Fracture resistance test was accomplished by subjecting the specimens to an axial compression force using a universal testing machine (Instron 3345, Canton, Massachusetts) to induce fracture. The vertical load was applied to the center of the occlusal table in a position close to that found clinically using 8-mm diameter metal sphere contacted the cuspal slopes of each specimen beyond the margins of the restorations at a crosshead speed of 0.5 mm/min until fracture occurred. The force required to make fracture was presented in Newton (N) [33, 44, 48].

To analyze the different failure patterns, the fractured specimens were examined under 40x magnification using stereomicroscope. The failure modes were classified as follows: cohesive fracture of the tooth (CS), adhesive fracture at the interface (AD), cohesive failure of the restorative material (CM), and complete fracture of the specimen (CO) [46].

### Statistical analysis

The obtained data were tabulated and coded using Microsoft Excel, then analyzed using the Statistical Package for the Social Sciences (IBM-SPSS, version 24, Armonk, NY, USA). At first, data was statistically checked for normality using Kolmogorov-Smirnov and Shapiro-Wilk tests (*p* > 0.05). Also, the equality of variance assumptions was checked with Levene test (*p* > 0.05). Since a normal distribution of the data and equality of variances had been confirmed, a parametric statistical procedure (2-way ANOVA) was used to test the significance of difference between group variability. The Tukey HSD post-hoc multiple comparison test was used to test the significance of difference between groups. The level of significance was set at p˂0.05.

## Results

The mean fracture resistance values (N) and the standard deviation for each group are displayed in Table 3. The 2-way ANOVA test indicated a significant difference among the groups regarding the restorative material and the storage time (*p* < 0.001).

**Table 3:** Means (Newton) and standard deviations (SDs) of fracture resistance for different groups (each, *n* = 10)

Groups	Mean (Newton) ± SD Immediate	Mean (Newton) ± SD Delayed
Group I, intact teeth	2969 ± 314.9 ^Aa^	2798 ± 295.8 ^Aa^
Group II, MOD prepared, unrestored teeth	1011 ± 128.6 ^Dd^	838.9 ± 136.0 ^Dd^
Group III, Filtek One Bulk Fill	2372 ± 262.6 ^Bb^	1713 ± 339.8 ^Bc^
Group IV, G-aenial bulk injectable	1719 ± 297.2 ^Cc^	1220 ± 148.5 ^Cd^
Group V, Neo Spectra ST LV	2304 ± 257.5 ^Bb^	1603 ± 301.9 ^Bc^

*Different uppercase letters indicate statistically significant differences among means of restored groups (columns)

**Different lowercase letters indicate statistically significant differences between means of storage time (rows)

(Tukey’s post-hoc test, *p* < 0.05)

For immediately tested groups, the results of Tukey HSD multiple comparison test revealed that intact teeth group had significantly the highest fracture resistance mean value among the other groups (*p* < 0.0001), while the prepared unrestored group had significantly the lowest fracture resistance mean value among all groups (*p* < 0.0001). Statistically significant difference was noted also between bulk-fill and no-cap flowable bulk-fill resin composite restored groups (*p* < 0.0001). Moreover, there was a statistically significant difference between conventional and no-cap flowable bulk-fill resin composite restored groups (*p* = 0.0001). However, no statistically significant difference was observed between bulk-fill and conventional resin composite restored groups (*p* = 0.9999).

For delayed tested groups, the results of Tukey HSD multiple comparison test revealed that intact teeth group had significantly the highest fracture resistance mean value among the other tested groups (*p* < 0.0001), while the prepared unrestored group had significantly the lowest fracture resistance mean value among all groups (*p* < 0.05). As well, statistically significant difference was noted between bulk-fill and no-cap flowable bulk-fill resin composite restored groups (*p* = 0.0026). Also, a statistically significant difference was found between conventional and no-cap flowable bulk-fill resin composite restored groups (*p* = 0.0493). Conversely, no statistically significant difference was observed between bulk-fill and conventional resin composite restored groups (*p* = 0.9947).

Regarding the effect of storage time, a statistically significant difference was found between the fracture resistance mean values of each immediate restored group and its counterpart of delayed groups (*p* < 0.05). Statistically significant difference was observed between immediate and delayed bulk-fill restored groups (*p* < 0.0001). Moreover, statistically significant difference was noted between immediate and delayed conventional resin composite restored groups (*p* < 0.0001). Also, there was a statistically significant difference between immediate and delayed no-cap flowable bulk-fill resin composite restored groups (*p* = 0.0022).

**Fig. 1:**
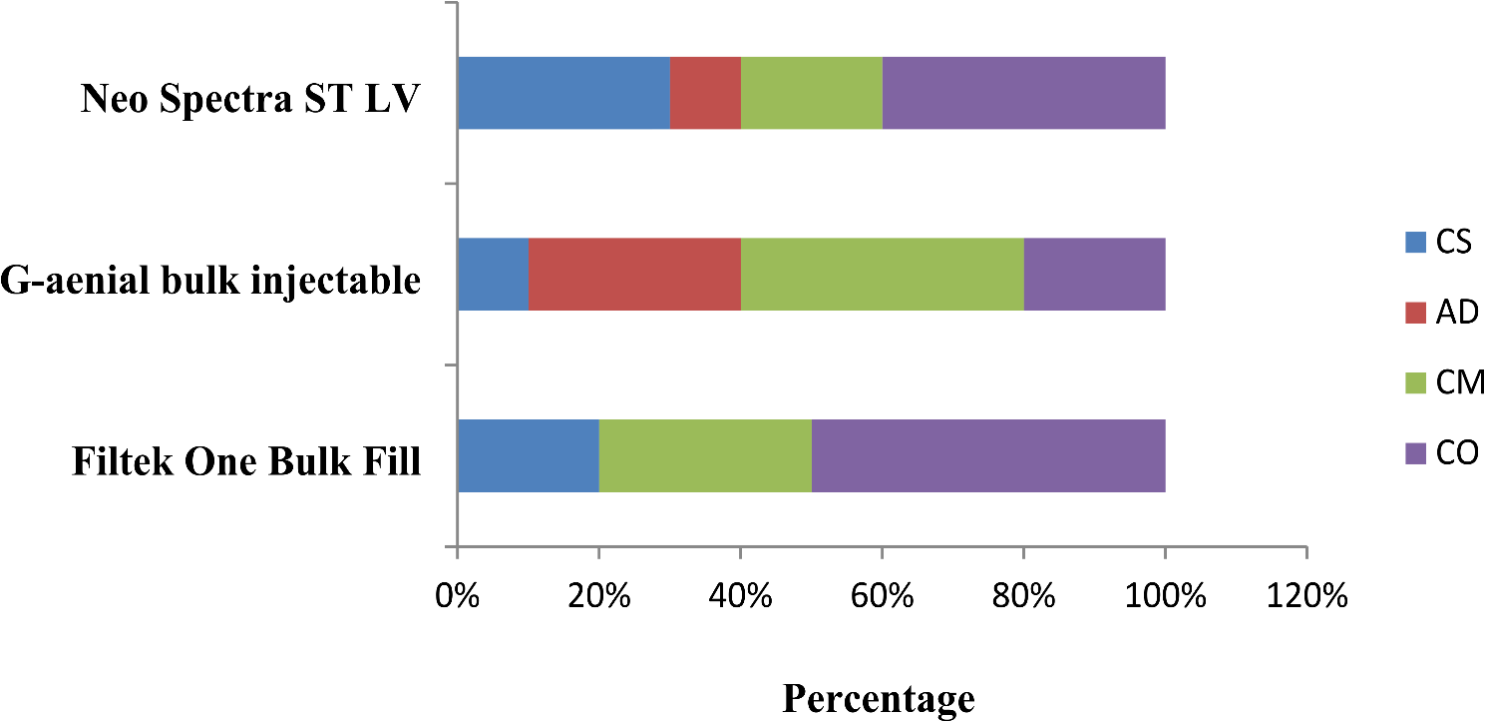
Percentage values of fractured patterns for immediate groups

**Fig. 2:**
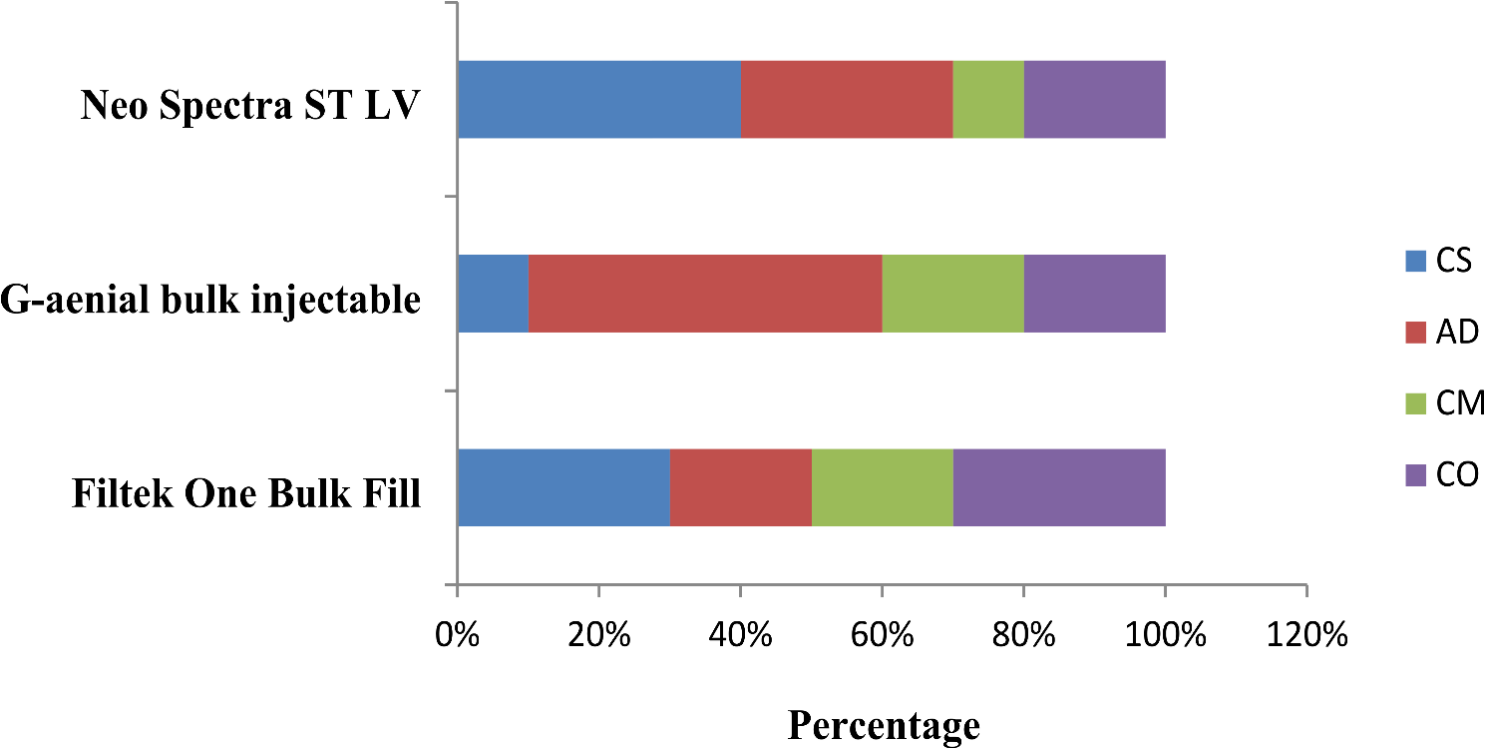
Percentage values of fractured patterns for delayed groups

**Fig. 3:**
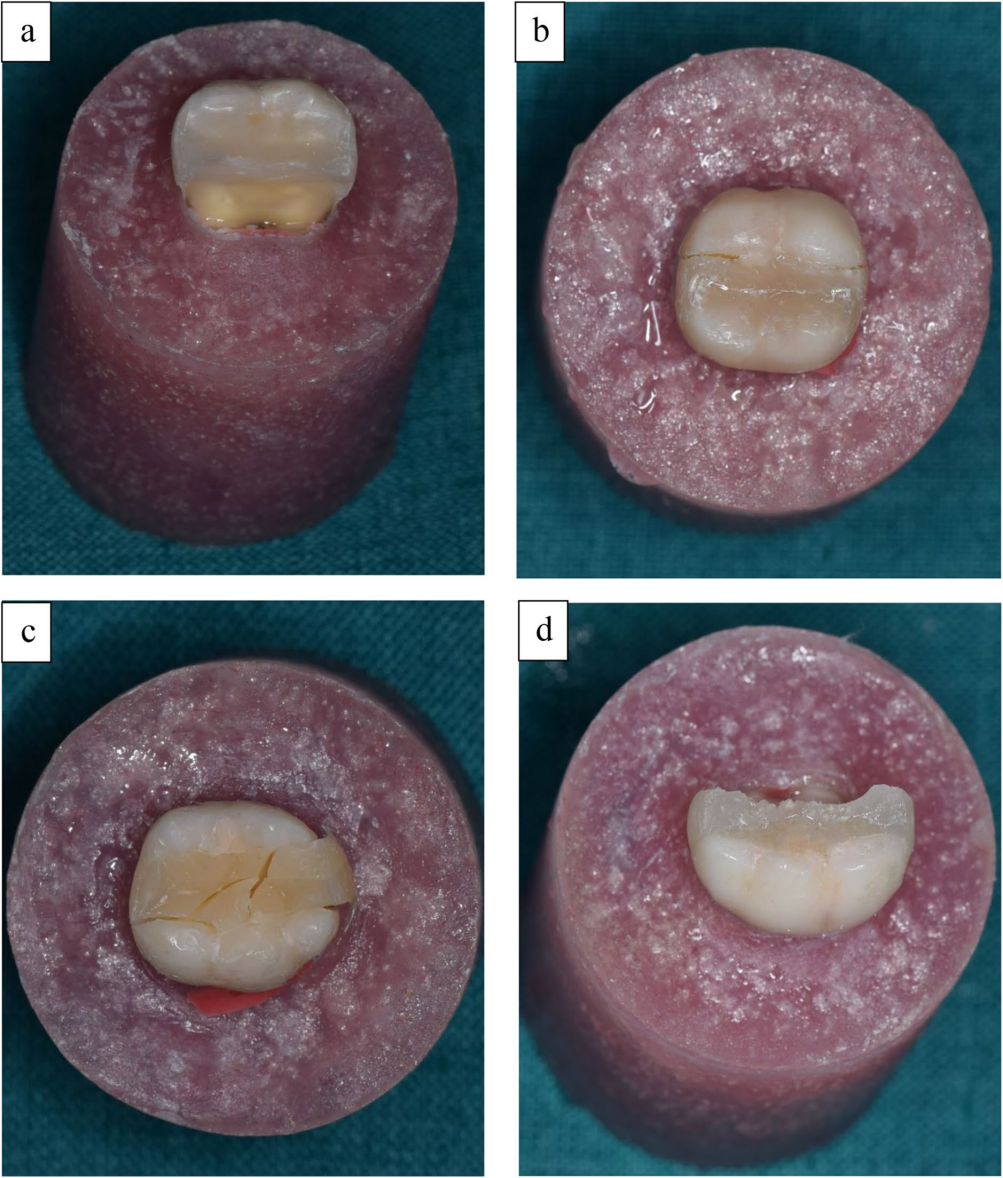
A representative photograph showing: **a**: cohesive fracture of the tooth, **b**: adhesive fracture at the interface between tooth and restoration, **c**: cohesive failure of the restoration, **d**: complete fracture of the specimen

The percentage values of fracture patterns for restored groups are presented in Figs. 1 and 2. The most frequent failure mode observed for immediate restored groups was complete fracture of the specimen involving the two cusps and restorative material followed by cohesive fracture of the restorative material. Cohesive fracture of the tooth and adhesive fracture at interface were the least observed. In delayed restored groups, the most frequent failure mode noted was adhesive failure at interface followed by cohesive fracture of the tooth, complete fracture of the specimen, and cohesive fracture of the restoration. Representative images of different fracture modes are shown in Fig. 3.

## Discussion

The optimal restorative procedures regarding the extensive carious lesions, tooth fracture, and unsatisfactory restorations are controversial. As direct restorations have the ability to strengthen the weakened remaining tooth structure, the compatibility in the physical and mechanical properties between the selected restorative material and the tooth is an essential factor for the success of restorative treatment. In the present in vitro study, the fracture resistance of molars with Class II MOD cavities restored with two different viscosities of bulk-fill resin composites were compared to those restored with conventional resin composite after 6-month water storage. Based on the study results, the null hypothesis formulated at the beginning of the study was rejected since a difference was found in the fracture resistance among different resin composite restored groups. Also, water storage had a negative influence on the tested groups.

The current study was conducted on molar teeth due to their higher susceptibility for prevalence of caries amongst the entire dentition in addition their exposure to heavy masticatory loads. The roots of the teeth were covered with an elastomeric impression material to mimic the periodontium. This coating material has the ability to undergo elastic deformation to avoid the stress concentrations in the cervical area of the tooth. The alveolar bone level was also simulated by embedding the selected molars vertically in acrylic resin cylinders 2 mm below C.E.J [49].

In this in vitro study, wide Class II MOD cavities were prepared in molar teeth to simulate the worst clinical situation that might face the dental practitioners due to the loss of both marginal ridges [50]. This cavity design also weakens the remaining tooth structure and favors cuspal fracture as a result of fatigue that caused by microcracks propagation under repeated occlusal loading which is suitable for measuring fracture resistance of restorative materials [7, 51]. The approximate standardization of cavity preparations was performed using a special device to avoid the variations in dimensions that might happened during preparations. This helped in preventing any procedural errors or incorrect explanation of the results, so any differences between tested groups would be ascribed to the effect of restorative materials [46].

The bulk-fill technique investigated in the present study represents one of the attempts during recent years that allows the clinicians to divert from using multiple increments to only one bulk increment when placing composite restorations in largecavities in order to simplify the procedure and reduce chair time [26, 48]. The newly introduced flowable bulk-fill composite is claimed to have satisfying features offering a single step application without final capping layer [52]. The incremental placement technique of conventional resin composite materials was also tested as it is considered the gold standard technique for restoring Class I and II cavities [53, 54]. Each tested material was used with its corresponding universal adhesive system as recommended by the manufacturer to achieve the best results. All adhesive systems were applied following selective enamel etching which is considered the most common bonding protocol used in everyday clinical practice [55].

It is very important when evaluating the performance of new restorative materials to duplicate the challenges that occurs in the oral cavity including temperature and humidity [42]. A total of 5000 thermal cycles were conducted following the regimen proposed by ISO standard (ISO TR 11450) representing temperature changes that may occur intraorally by hot and cold extremes [38]. For more simulation of oral environment and further increase of the aging effect, half of the specimens was stored in distilled water for 6 months which provoked water sorption and elution of suboptimal polymerized monomers [56]. Moreover, water storage might significantly impact the overall restoration quality [57].

Resin composite materials and dental adhesives have been significantly improved over the past decade aiming to reinforce the remaining tooth structure and providing adequate strength in order to withstand the forces of mastication [20]. Conducting in vitro studies that aim to examine the fracture resistance of restored teeth is a crucial method for improving the restorative procedures [43, 44, 46]. The fracture resistance is influenced by many factors including the method of tooth fixation, the type of load application device, and the speed of crosshead [58]. To minimize the diversity between clinical and laboratory assessments, the joint use of mechanical tests and failure mode analysis were used [59, 60]. In the current study, a standardized method was utilized for assessing the fracture resistance by applying a metal sphere with 8-mm diameter to the slopes of the cusps and in the center of the occlusal table in a position close to that found clinically [48].

For both immediate and delayed tested groups, the study results showed that intact teeth group had the highest fracture resistance mean value among the other groups. This could be attributed to the presence of continuous circle of tooth structure including intact cusps and marginal ridges. This helped in strengthening and maintaining the tooth integrity [61, 62]. However, the lowest fracture resistance mean value was noted in the prepared unrestored teeth group. This could be ascribed to the weakening effect of MOD cavity preparation that reduced the structural integrity of the tooth with more susceptibility to fracture. Moreover, the compressive load application on unrestored teeth resulted in a wedging effect between buccal and lingual cusps leading to a decrease in their fracture resistance [20, 44]. This was in agreement with previous studies [45, 59] which reported a reduction in the fracture resistance of teeth that had received extensive preparations.

Resin composite restored groups did not show any significant improvement in the fracture resistance of prepared teeth when compared to intact group as removal of large amounts of tooth structure during cavity preparation and the increase in cavity width played a crucial role in decreasing the fracture strength of the restored tooth. Thus, could not withstand heavy compressive load [63, 64]. However, it was noted that all immediate and delayed restored groups exhibited improvements in the fracture resistance when compared to prepared unrestored group irrespective of the type of resin composite material used. This could be attributed to the capability of resin composite in transmitting and distributing the functional stresses through the tooth-restoration interface owing to the resin interlocking with dentin and hybrid layer formation with the ability to reinforce the remaining tooth structure [65, 66].

Based on the finding of the current study, there was no difference in the fracture resistance of immediate bulk-fill and conventional resin composite restored groups. As well, the same result was obtained after 6-month water storage. This could be related to the similarity in filler loading between Filtek One Bulk Fill (76.5 wt%) and Neo Spectra ST LV (76–78 wt%). Filtek One bulk-fill incorporates two methacrylate monomers; high molecular weight aromatic urethane dimethacrylate (AUDMA) and addition fragmentation monomer (AFM). These monomers help in decreasing the volumetric shrinkage and providing relaxation mechanism with subsequent stress relief [20, 35]. This is consistent with the lower shrinkage tendency of Neo Spectra ST LV which is based primarily on an optimized resin matrix with methacrylate modified polysiloxane that could result in reducing the polymerization shrinkage stresses [33, 67]. Moreover, both resin composite materials were manufactured based on nanofiller technology. This outcome is supported by the results of previous studies [20, 45, 68] which revealed that high-viscosity bulk-fill nanocomposite showed fracture resistance values similar to the incrementally placed composite. On the contrary, Leprince et al. [69] and Ilie et al. [22] reported that bulk-fill resin composites displayed inferior mechanical characteristics when compared to incrementally placed ones.

The overall results for immediate and delayed restored groups showed that the recently introduced G-aenial bulk injectable resin composite could not restore the original tooth fracture resistance as the extended width of the occlusal isthmus played an essential role in reducing teeth strength. This could be related to its lower filler content and the similar polymerization shrinkage to the conventional flowable resin composite as stated in a previous report [37]. Jang et al. [70] showed that flowable bulk-fill composites had higher polymerization shrinkage when compared to conventional resin composites. Alshehri et al. [71] also stated that newer class of flowable bulk-fill resin composite couldn’t be used without covering layer in posterior teeth due to its inferior physico-mechanical properties. However, Franca et al. [72] reported a contradictory result where no difference was observed in the fracture strength of teeth restored with conventional or bulk-fill resin composites of different viscosities.

The study results indicated a significant variability in the fracture resistance of the specimens. The material with higher fracture resistance had the ability to resist the formation and propagation of microcracks [38, 40]. The difference in fracture strength between resin composite groups might be attributed to the variation in their resin matrix system, filler content, filler size, and distribution. Thus, the decrease in the size of fillers with an increase in their load resulted in increasing the compressive strength of the material [73].

Storage of resin composite groups for 6 months resulted in a significant decrease in their fracture resistance. This could be attributed to water sorption into resin composite restorations that can result in impairing the polymer network, degradation of the resin matrix, and hydrolysis of the filler-matrix interface [74]. Previous studies [75, 76] reported that artificial aging had an impact on leaching of particular resin composite filler particles. Whilst, Drummond et al. [77] assumed that the deterioration of resin composite was related more to degeneration of the resin-matrix and debonding of filler-matrix than degradation of the glass fillers. The result of the study was in congruence with Coelho-De-Souza et al. [57] who stated that the prolonged aging led to significant decrease in fracture resistance of MOD resin composite restorations. In addition, Jang et al. [70] reported that conventional resin composites are less prone to water deterioration that flowable bulk-fill composites.

Fractured patterns of restored groups were analyzed to predict the probability of failure that might occur for each tooth-restoration complex and its clinical longevity. The predominant failure mode for immediate restored groups was catastrophic fracture of the specimens. This could be explained by the lower cuspal strains and shrinkage stresses generated by Filtek One Bulk Fill and Neo Spectra ST LV resin composite restorations. Moreover, those observed severe fractures were in accordance with what might happen in the oral environment. Van Ende et al. [78] reported that materials with low polymerization shrinkage stresses were often associated with complete fracture of tooth and restoration. Also, the cohesive failure of restoration was frequently observed which could be related to the normal stress distribution during the fracture resistance test [79]. The cohesive fracture of tooth structure was noted mainly in the lingual cusp. This could be ascribed to the lower structural volume of lingual cusp than the buccal cusp in addition to the higher incidence of cracks observed for these cusps [43].

After 6-month water storage, the most frequent failure mode observed was the adhesive failure at the interface between the tooth and restoration. This could be related to the hydrolytic degeneration of adhesive system as a result of slow water hydrolysis weakening the bond strength and promoting adhesive breakdown. This result was in agreement with a previous study [57] which showed an increase in adhesive failure for resin composite restorations after 6 months of storage in artificial saliva.

In spite of the attempts that performed to simulate the oral environment, the outcome of this in vitro study has certain limitations. The specimens were subjected to compressive loading to induce fracture without prior cyclic loading. Thus, more studies are necessary to assess the resistance of resin composite materials after dynamic loading as it is more clinically relevant. Another limitation of the current study is the use of static loading where the force was slowly applied with crosshead speed of 0.5 mm/min. This corresponds to the load in a parafunctional condition rather than to normal occlusal type load. Therefore, more relevant test methods should be established to mimic what occurs clinically. Despite such limitations, the results of this study provided helpful data on the newly introduced no-cap flowable bulk-fill composite. However, further clinical studies are needed to fully assess its biomechanical resistance.

## Conclusions

Within the limitations of this current study, the following conclusions can be drawn:

- The restored molar teeth had inferior fracture resistance compared to intact teeth.
- Under compressive loads, there was no difference in the fracture resistance of Class II MOD cavities restored with bulk-fill or conventional resin composites. However, both materials showed advantageous fracture resistance in comparison to no-cap flowable bulk-fill resin composite.
- Six-months water storage had negatively affected the fracture resistance of molar teeth restored with different resin composite restorative systems.

## Data Availability

The datasets used and/or analyzed during the current study are available from the corresponding author on reasonable request.

## Abbreviations

MOD: Mesio-occluso-distal
LED: Light-emitting diode
AFM: Addition fragmentation monomer
AUDMA: Aromatic urethane dimethacrylate
UDMA: Urethane dimethacrylate
DDDMA: 1,12-dodecane dimethycrylate
EDMAB: Ethyl 4-dimethyl aminobenzoate
YbF3: Ytterbium fluoride
MDP: Methacryloyloxydecyl dihydrogen phosphate
HEMA: Hydroxyethyl Methacrylate
Bis-EMA: Ethoxylated bisphenol-A dimethacrylate
UV-light: Ultraviolet light
4-MET: 4-methacryloxyethyl trimellitate
MDTP: 10-methacryloyloxydecyl dihydrogen thiophosphate
BHT: Butylated hydroxytoluene
PENTA: Dipentaerythritol pentacrylate phosphate
C.E.J: Cemento-enamel junction
